# Supplementary material for: Influence of substrate types and morphological traits on movement behavior in a toad and newt species
Source: PeerJ. 2019 Jan 9;6:e6053. doi: 10.7717/peerj.6053 (PMC6330043; doi:10.7717/peerj.6053)
Supplement: Supplemental Information 2 — ***: P < 0.001, **: P < 0.01. +: significant positive effect of the factorial variable. [file peerj-07-6053-s002.docx]

Supplementary Material. Best linear mixed-effects models (in bold) retained by AIC selection showing the significant variables having effect on the crossing speed (in cm/s) for the marbled newt (*Triturus marmoratus*; N = 20) and the common toad (*Bufo bufo*; N = 77). *leg*: relative hind-limb length. *stops*: number of stops. ***: P < 0.001, **: P < 0.01. +: significant positive effect of the factorial variable.

|  | Intercept | Species | Substrate | *leg* | *stops* | Species*Substrate | Df | logLik | AICc | ∆AIC |
| --- | --- | --- | --- | --- | --- | --- | --- | --- | --- | --- |
| **Model 1** | **2.261** | **+** | **+** | **0.999** | **-0.251** |  | **7** | **18.846** | **-23.5** | **0.00** |
| **Model 2** | **2.308** | **+** | **+** |  | **-0.255** |  | **6** | **17.347** | **-22.5** | **0.95** |
| Model 3 | 2.269 | + | + | 0.101 | -0.249 | + | 8 | 18.795 | -21.3 | 2.16 |
